# Draft genome sequence of *Dolosicoccus paucivorans* DSM 15742^T^ isolated from a patient’s blood in Cleveland, Ohio

**DOI:** 10.1128/mra.01296-24

**Published:** 2025-05-28

**Authors:** Angelina Garcia Guzman, Andrea Orozco, Haleema Amin, Rüdiger Pukall, Markus Goeker, Natalia Ivanova, Rekha Seshadri, Tricia A. Van Laar

**Affiliations:** 1 Department of Biological Sciences, California State University, Stanislaus 14674, Turlock, California, USA; 2 Leibniz Institute DSMZ-German Collection of Microorganisms and Cell Cultures GmbH, Braunschweig, Germany; 3 DOE Joint Genome Institute, Lawrence Berkeley National Laboratory 1666, Berkeley, California, USA; University of Maryland School of Medicine, Baltimore, Maryland, USA

**Keywords:** pathogen

## Abstract

This study describes the whole genome sequence of *Dolosicoccus paucivorans* 2991-95 (DSM 15742) isolated from human blood in Cleveland, Ohio. The genome length is approximately 1.8 Mbp with 107 contigs and a G + C content of 37.91%. Annotation identified 1,752 coding genes and multiple virulence factors.

## ANNOUNCEMENT

In 1995, a 78-year-old male was admitted to the hospital in Cleveland, Ohio, with symptoms of pneumonia. He was found to have a previously uncharacterized catalase-negative, α-hemolytic, gram-positive coccus in his bloodstream (1). The organism was unidentifiable by the Streptococcus Laboratory at the Centers for Disease Control and Prevention at the time of collection, but 16S rRNA sequencing and analysis (GenBank accession number AJ012666) and biochemical testing identified this as a new organism that was named *Dolosicoccus paucivorans* 2991-95 (DSM 15742^T^) (1). Other isolates of this species isolated from humans have been identified in the cervix and as part of the urinary microbiome (2), though *D. paucivorans’* role in pathogenesis is unclear.

This organism was selected for sequencing as part of the Genomic Encyclopedia of Bacteria and Archaea) project (3, 4). The major thrust of this project was to sequence genomes of organisms that represent new phylogenetic clades. *D. paucivorans* DSM 15742^T^ is the type strain and first representative of its genus. For DNA isolation, the bacterium was grown on DSMZ medium 92 (https://mediadive.dsmz.de) for 24 hours at 37°C at the Leibniz Institute DSMZ. The DNA was then isolated using the Masterpure Gram-positive DNA Extraction Kit (Epicentre) and sent to the DOE Joint Genome Institute (JGI), where an Illumina short-insert paired-end library with an average insert size of 300 bp was constructed and sequenced on the Illumina HiSeq 2500 sequencer using HiSeq TruSeq SBS sequencing kits, v4, following a 2 × 150 indexed run recipe. Raw Illumina sequence data were passed through the filtering program BBDuk (5). Filtered reads were assembled with Velvet (version 1.2.07) (6) to create Velvet contigs, from which paired-end reads were simulated using wgsim (version 0.3.0) (7). Contigs and simulated read pairs were assembled using Allpaths-LG (version r46652) (8). The following parameters were used: Velvet (velveth: 63 –shortPaired and velvetg: –very clean yes –exportFiltered yes –min contig lgth 500 –scaffolding no –cov cutoff 10) 2) wgsim (–e 0-1 100-2 100 –r 0 –R 0 –X 0) 3) Allpaths–LG (PrepareAllpathsInputs:, RunAllpathsLG:). The total number of raw reads was 11,416,440, and the total amount of DNA sequenced was 1,723.9 Mbp. Genome completeness was assessed using CheckM2 version 1.0.2 (9), which found that the genome was 96.89% complete with 0.16% contamination. Genome annotation was performed with the JGI Microbial Genome Annotation Pipeline version 4 (10) using the standard DOE-JGI protocol.

**Fig 1:**
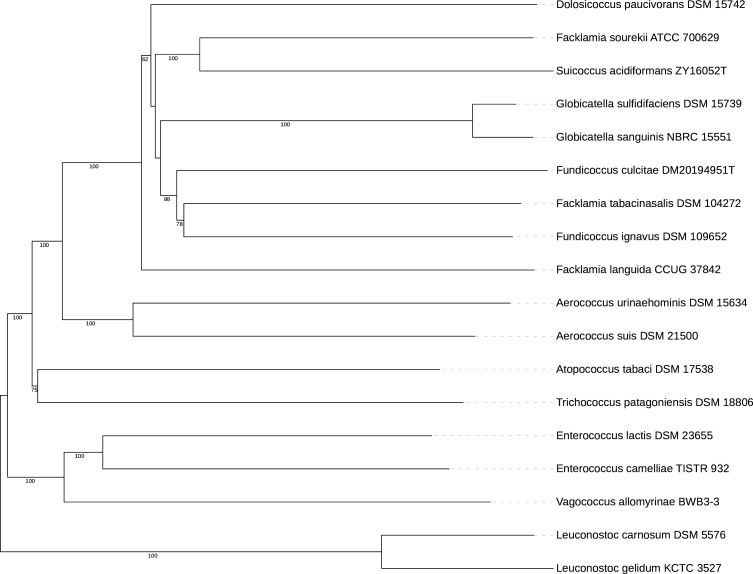
Tree inferred with FastME 2.1.6.1 (15) from whole proteome-based Genome BLAST Distance Phylogeny (GBDP) distances. The branch lengths are scaled via the GBDP distance formula *d_5_*. Branch values are GBDP pseudo-bootstrap support values > 60% from 100 replications, with an average branch support of 87.7%. The tree was midpoint rooted (16).

The genome of *D. paucivorans* is 1,759,599 bp with a G + C content of 37.91%. There are 107 contigs with an *N*_50_ of 26.3 kb. Annotation identified 1,752 protein-coding genes, 35 tRNAs, and 3 rRNAs (Table 1). Using dDDH-d4 values, the Type Strain Genome Server (11) identified the closest relatives of *D. paucivorans* as *Leuconostoc gelidum* (38.2%), *Vagococcus allomyrinae* (34.4%), and *Leuconostoc carnosum* (Fig. 1). We used PathogenFinder version 1.1 (12) to predict a probability of 0.937 of *D. paucivorans* as a human pathogen. Using the core data set B from the Virulence Finder Database (13), we identified some virulence factors, including genes for the production of adhesins, capsule/immune modulation proteins, stress response, and iron sequestration. We used ResFinder version 4.5.0 (14) to search for potential antimicrobial resistance cassettes and identified the presence of a gene (*aadD1*) for aminoglycoside resistance and one for bleomycin resistance (*bleO*). The genome also contains one CRISPR locus.

**TABLE 1:** Genome features of *D. paucivorans* DSM 15742^T^

Feature	Finding
Total scaffold sequence length (bp)	1,759,599
No. of contigs	107
Contig *N*_50_ (kbp)	26.3
Genome coverage	776×
GC content (%)	37.91
No. of genes	1,800
No. of protein-coding genes	1,752
No. of rRNA	3
No. of tRNA	35

## Data Availability

The genome sequence was deposited in GenBank under the accession number FNEL00000000. The raw sequences have been deposited in the NCBI SRA under the accession number SRS1670301. Additional data can be explored and downloaded from the IMG/M portal using the taxon ID 2622736585.
